# Detection of the Omicron BA.1 Variant of SARS-CoV-2 in Wastewater From a Las Vegas Tourist Area

**DOI:** 10.1001/jamanetworkopen.2023.0550

**Published:** 2023-02-23

**Authors:** Van Vo, Richard L. Tillett, Katerina Papp, Ching-Lan Chang, Anthony Harrington, Michael Moshi, Edwin C. Oh, Daniel Gerrity

**Affiliations:** 1 Laboratory of Neurogenetics and Precision Medicine, University of Nevada Las Vegas; 2 Nevada Institute of Personalized Medicine, University of Nevada Las Vegas; 3 Southern Nevada Water Authority, Las Vegas; 4 UNLV School of Medicine, Department of Internal Medicine, University of Nevada Las Vegas

## Abstract

**Importance:**

Interpretation of wastewater surveillance data is potentially confounded in communities with mobile populations, so it is important to account for this issue when conducting wastewater-based epidemiology (WBE).

**Objectives:**

To leverage spatial and temporal differences in wastewater whole-genome sequencing (WGS) data to quantify relative SARS-CoV-2 contributions from visitors to southern Nevada.

**Design, Setting, and Participants:**

This cross-sectional wastewater surveillance study was performed during the COVID-19 pandemic (March 2020 to February 2022) and included weekly influent wastewater samples that were analyzed by reverse transcription–quantitative polymerase chain reaction to quantify SARS-CoV-2 RNA and WGS for identification of variants of concern. This study was conducted in the Las Vegas, Nevada, metropolitan area, which is a semi-urban area with approximately 2.3 million residents and nearly 1 million weekly visitors. Samples were collected from 7 wastewater treatment plant (WWTP) locations that collectively serve the vast majority of southern Nevada (excluding the small number of septic systems) and 1 manhole serving the southern portion of the Las Vegas Strip. With Las Vegas tourism returning to prepandemic levels in 2021, it was hypothesized that visitors were contributing a disproportionate fraction of SARS-CoV-2 RNA to the largest WWTP in southern Nevada, potentially confounding efforts to estimate COVID-19 incidence in the local community through WBE.

**Main Outcomes and Measures:**

Relative SARS-CoV-2 load and variants from visitors vs the local population.

**Results:**

The Omicron BA.1 VOC was detected in the Las Vegas Strip manhole approximately 1 week before its detection at the WWTP locations (December 13, 2021) and by clinical testing (December 14, 2021). On December 13, Omicron-specific mutations represented a mean (SD) of 48.0% (4.2%) of all genomes from the Las Vegas Strip manhole and 4.1% (1.4%) of all genomes at facilities 2 and 3; by December 20, Omicron-specific mutations represented means (SD) of 82.0% (3.0%) of all genomes at the Las Vegas Strip manhole and 48.0% (2.8%) of all genomes at facilities 2 and 3, respectively. During this time, it was estimated that visitors contributed more than 60% of the SARS-CoV-2 load to the sewershed serving the Las Vegas Strip and that Omicron prevalence among visitors was 40% to 60% on December 13 and 80% to 100% on December 20th.

**Conclusions and Relevance:**

Wastewater surveillance is a valuable complement to clinical tools and can provide time-sensitive data for decision-makers and policy makers. This study represents a novel approach for quantifying the confounding effects of mobile populations on wastewater surveillance data, thereby allowing for modification of an existing WBE framework for estimating COVID-19 incidence in southern Nevada.

## Key Points

**Question:**

Does tourism explain the disproportionate increase in SARS-CoV-2 load observed in the Las Vegas Strip sewershed, and can this contribution be quantified to more accurately estimate COVID-19 incidence in the local community?

**Findings:**

In this cross-sectional study, the rapid onset of the Omicron BA.1 variant of concern in Las Vegas Strip wastewater was leveraged to quantify relative SARS-CoV-2 contributions from visitors (>60%) and estimate Omicron prevalence in this subpopulation in late 2021 (40%-60% on December 13 and 80%-100% on December 20).

**Meaning:**

These findings suggest that mobile populations (eg, tourists and commuters) may disproportionately affect wastewater surveillance data, leading to overestimates of infection burden with wastewater-based epidemiology.

## Teaser

This cross-sectional study leverages spatial and temporal differences in wastewater whole-genome sequencing data to quantify relative SARS-CoV-2 contributions from visitors to southern Nevada.

## Introduction

Recent studies demonstrate that municipal sewage can be monitored to assess COVID-19 incidence and evaluate community transmission of variants of concern (VOCs).^1,2,3,4^ However, interpretation of wastewater surveillance data are potentially confounded in communities with mobile populations, including tourists and commuters. Studies show that mobile populations can have a significant impact on wastewater flow rate^3^ and contaminant loading,^5^ particularly during special events and on holidays and weekends.^6,7^ The implications for wastewater-based epidemiology (WBE) have not been adequately assessed, so it is unclear how this issue might affect wastewater-derived estimates of COVID-19 incidence or other public health metrics.

In March 2020, Nevada’s stay-at-home directive forced the closure of all hotels and casinos along the Las Vegas Strip—an economic corridor known to attract nearly 1 million weekly visitors prior to the COVID-19 pandemic.^8^ As a result, wastewater flows in the sewershed decreased by approximately 15%,^3^ highlighting visitor impacts on operations at the corresponding wastewater treatment plant (WWTP). This was actually fortuitous because it provided a unique opportunity to develop a WBE framework for estimating COVID-19 incidence in southern Nevada without the confounding effects of visitors.^4^ However, the validity of this initial framework was called into question as business and tourism returned to prepandemic levels, specifically due to uncertainty in estimating SARS-CoV-2 loads from visitors. Due to heightened COVID-19 awareness and the fact that international travelers to the United States required a negative diagnostic test (lifted as of June 2022), COVID-19 prevalence may have been lower among visitors relative to the general population,^9^ at least prior to travel,^10^ but this required confirmation for continued use of the existing WBE framework.

In this study, we quantified and sequenced SARS-CoV-2 RNA in sewage from a manhole near the Las Vegas Strip (visitor dominated), the WWTP serving the Las Vegas Strip and the surrounding community (visitors and locals), and all other WWTPs in southern Nevada (local dominated). We then asked whether temporal and spatial differences, specifically related to prevalence of the Delta and Omicron variants, could help determine the relative SARS-CoV-2 load from visitors as a means of generating a more accurate estimate of COVID-19 incidence in the local population. If successful, this would represent a novel approach for understanding the potential confounding effects of mobile populations in WBE applications.

## Methods

### Wastewater Surveillance

The University of Nevada Las Vegas (UNLV) Institutional Review Board reviewed this project and determined it to be exempt from human participant research, including the requirement for informed consent, according to federal regulations and university policy. UNLV’s Institutional Biosafety Committee also approved the methods and techniques used in this study. Our study follows the Strengthening the Reporting of Observational Studies in Epidemiology (STROBE) reporting guidelines.^11^

**Figure 1.**
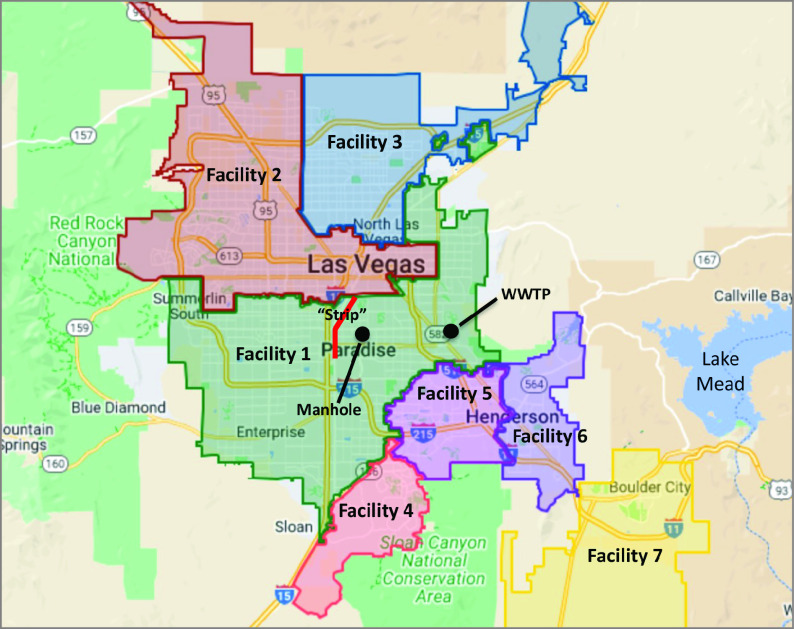
Map of the Las Vegas Metropolitan Area and Its Sewershed Boundaries The map illustrates general locations of the Las Vegas Strip, the manhole sampling location for this study, and the facility 1 wastewater treatment plant (WWTP). Samples collected from the manhole are largely representative of sewage from the southern portion of the Las Vegas Strip (ie, visitor dominated). The manhole and the entirety of the Las Vegas Strip are located within the facility 1 sewershed (ie, visitors and locals), while other sewersheds are more representative of the local population. Adapted from Google Maps.

Wastewater surveillance in southern Nevada was initiated in March 2020 and now includes 7 weekly samples from local WWTPs serving the approximately 2.3 million residents of the Las Vegas metropolitan area (Figure 1). For this study, we also monitored a sewer manhole serving the southern portion of the Las Vegas Strip (Figure 1). Collectively, these locations yield samples that are visitor dominated (manhole), local dominated (facilities 2-7), and a blend of locals and visitors (facility 1). Using previously published methods,^3,4^ SARS-CoV-2 RNA was quantified in these samples with reverse transcription–quantitative polymerase chain reaction assays for 4 genomic targets (ie, *orf*1a, E, N1, N2). After adjusting for equivalent sample volume and recovery of spiked bovine coronavirus, final concentrations were reported in gene copies per liter (gc/L).

For sequencing, viral RNA was extracted from wastewater using the AllPrep PowerViral DNA/RNA Kit (Qiagen) and the Wizard Enviro TNA Kit (Promega). The nucleic acid was eluted in 100 μL of RNase-free dH_2_O, any residual DNA was digested using DNase I, and the RNA was purified using the RNeasy PowerClean Pro Cleanup Kit (Qiagen). The final RNA extract was eluted in RNase-free dH_2_O, and more than 10 ng of purified RNA was processed for first-strand cDNA synthesis. Whole-genome sequencing (WGS) libraries were constructed using the CleanPlex SARS-CoV-2 FLEX Panel (Paragon Genomics) according to manufacturer’s instructions. Libraries were sequenced using an Illumina NextSeq 500 platform and a midoutput version 2.5 (300 cycles) flow cell. Illumina adapter sequences were trimmed from reads using cutadapt version 3.2. All sequencing reads were mapped to the SARS-CoV-2 genome (NC_045512.2) using bwa mem version 0.7.17-r1188. Amplicon primers were trimmed from aligned reads using fgbio TrimPrimers version 1.3.0, and variants were called with iVar variants version 1.3. Genome coverage (>95% with a median depth of >100-fold) was calculated by samtools coverage version 1.10. The 19 characteristic mutations used to identify Omicron BA.1 and their frequencies of detection are summarized in eTable 1 in Supplement 1.

### Statistical Analysis

SARS-CoV-2 RNA concentrations were converted to viral loads using the trapezoidal rule coupled with average daily flow rates for each WWTP.^4^ Viral loads were subsequently converted to COVID-19 incidence using a previously determined shedding parameter calibrated for southern Nevada (11.4 log_10_ gc/infection).^4^

**Figure 2.**
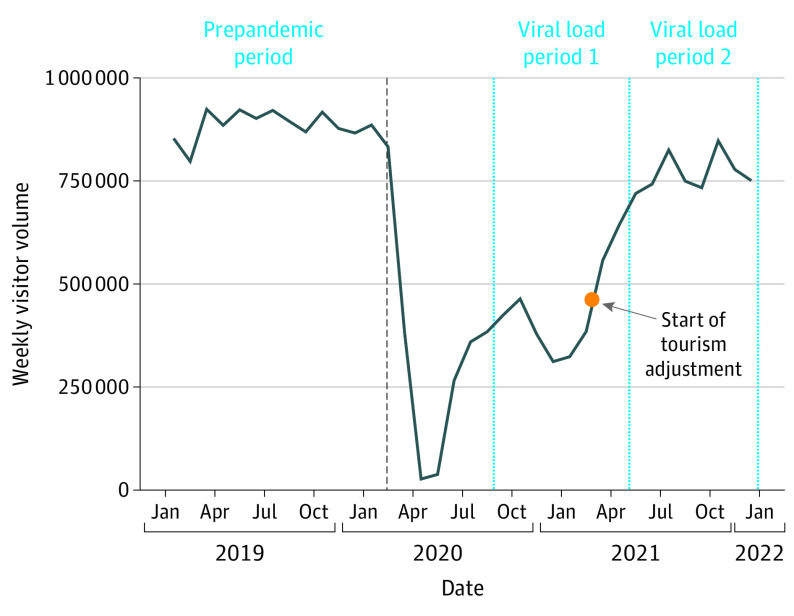
Las Vegas Weekly Visitor Volume for 2019-2021 Dashed blue lines delineate the two 8-month time periods for which wastewater SARS-CoV-2 loads were compared across the wastewater treatment plants.

**Figure 3.**
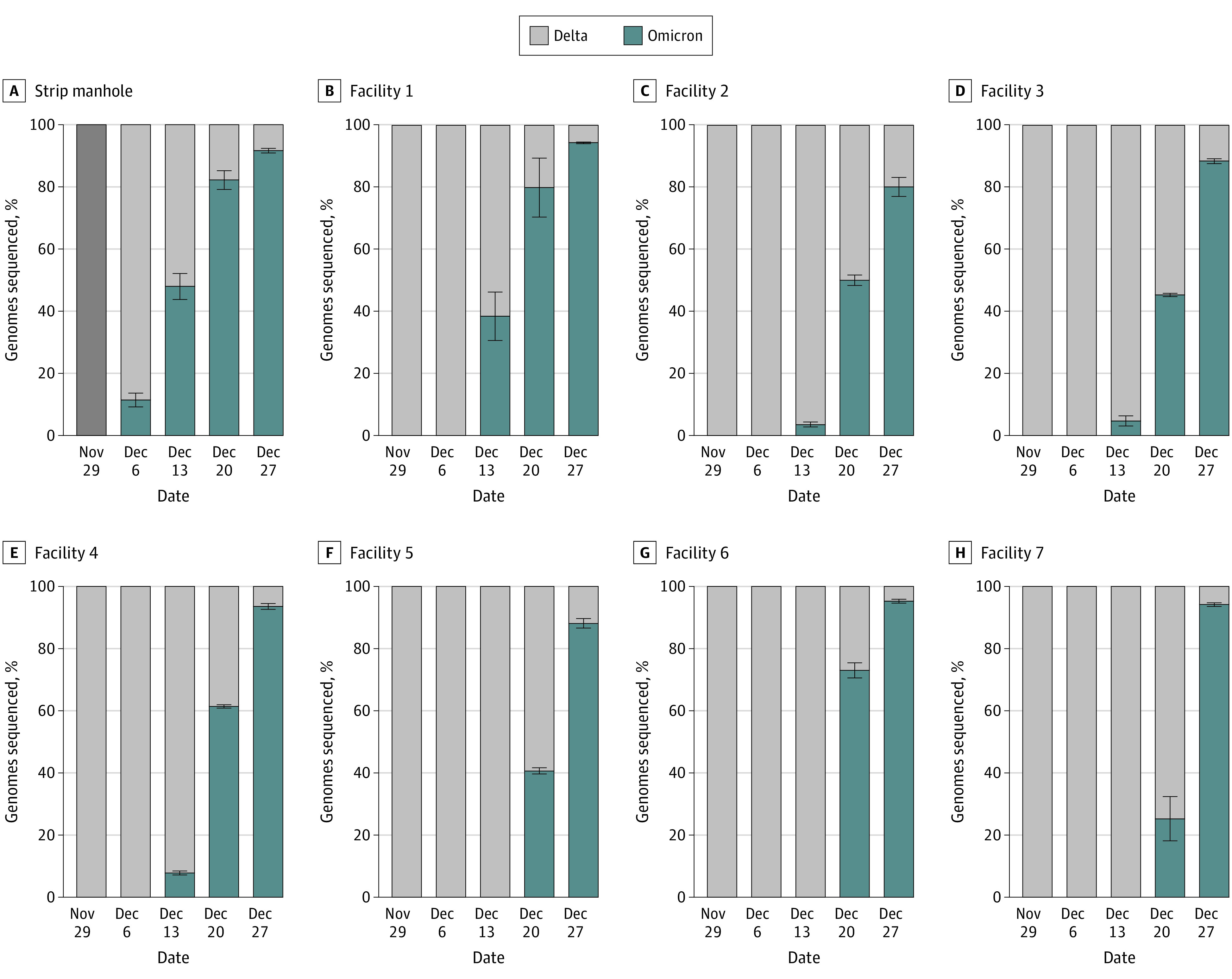
Wastewater Prevalence of Delta and Omicron BA.1 Variants in Southern Nevada, Determined by Whole Genome Sequencing of Wastewater Samples From the Las Vegas Strip Manhole and Facilities 1-7. No sample was obtained from the Las Vegas Strip on November 29.

In March 2020, a stay-at-home directive shut down operations along the Las Vegas Strip, leading to an immediate decline in weekly visitors (Figure 2). Upon reopening, weekly visitor volume briefly increased in June 2020, declined due to a COVID-19 surge in the winter of 2020 to 2021, and then approached prepandemic levels by the summer of 2021 (Figure 2). To eliminate the confounding effect of visitors on wastewater-derived COVID-19 incidence estimates, the viral load at facility 1, which receives flows from the Las Vegas Strip, was adjusted downward by 60% starting in March 2021. As described later, this value was determined by incorporating wastewater sequencing data for 2 dates in December (Figure 3) into the following mass balance equation:

Visitor Relative Load × Omicron Prevalence Among Visitors + (1 − Visitor Relative Load) × Omicron Prevalence at Facilities 2 and 3 = Omicron Prevalence at Facility 1.

## Results

WGS of local wastewater identified early VOCs (eg, Alpha and Epsilon) up to 1 month before their confirmation in clinical samples.^4^ Similarly, Omicron BA.1 was first detected in wastewater on December 7, 2021, at the Las Vegas Strip manhole (Figure 3A), but the first local Omicron BA.1 infection was not clinically confirmed until December 14, 2021.^12^ Omicron-specific mutations were first detected at local WWTPs on December 13 (mean [SD] frequency, manhole: 48.0% [4.2%]; facility 1: 38.0% [7.8%]; facilities 2-3: 4.1% [1.4%]) and then represented more than 80% of all wastewater genomes by December 27 (mean [SD] frequency, manhole: 82.0% [3.0%]; facility 1: 80.0% [9.5%]; facilities 2-3: 48.0% [2.8%]) (Figure 3B-H), highlighting the rapid displacement of the Delta VOC. Over a similar timeframe, wastewater SARS-CoV-2 concentrations increased at least 10-fold across southern Nevada, mirroring the surge in confirmed cases (eFigure in Supplement 1).

During 2 consecutive 8-month time periods (September 2020 to April 2021 and May to December 2021), the WWTP serving the Las Vegas Strip (facility 1) received 32% and 68% of its total viral load, respectively, thereby suggesting a considerable increase in late 2021 (Table). In contrast, the 2 most similar sewersheds in terms of population and infection burden (facilities 2 and 3) received approximately 50% of their total viral loads in each period (Table), and all 3 sewersheds exhibited similar mean (SD) ascertainment ratios earlier in the pandemic (3.4 [0.4]).^4^ The ascertainment ratio represents the estimated undercount of COVID-19 cases and is calculated as the wastewater-derived infection estimate divided by the sewershed-specific confirmed case count. By late 2021, the mean (SD) ascertainment ratio for facility 1 (14.0 [4.8]) had deviated considerably from facilities 2 (4.2 [1.4]) and 3 (4.5 [1.2]) (eTable 2 in Supplement 1). We also observed more rapid onset of Omicron-specific mutations at the Las Vegas Strip manhole and facility 1 (Figure 3A and B) than at facilities 2 to 7 (Figure 3C-H). We hypothesized that with tourism returning to prepandemic levels (Figure 2), visitors started contributing a disproportionate fraction of SARS-CoV-2 RNA to facility 1, and in early December 2021, these visitors were more likely to be infected with the Omicron VOC than the local population.

**Table.** Summary of Facility-Specific Wastewater SARS-CoV-2 Loads for 2 Consecutive 8-Month Time Periods, September 1, 2020, to December 31, 2021^a^

Facility	Population	Proportion of total, %	Flow rate, mg/d	September 1, 2020, to April 30, 2021			May 1 to December 31, 2021			Site total
				Viral load, gc	Site total, %	Period total, %	Viral Load, gc	Site total, %	Period total, %	Viral load, gc
**Before tourism adjustment**										
1	872 009	39	100	6.33 × 10^16^	32	41	1.36 × 10^17^	68	62	1.99 × 10^17^
2	757 418	34	42	4.55 × 10^16^	54	30	3.83 × 10^16^	46	17	8.39 × 10^16^
3	255 008	11	20	1.68 × 10^16^	52	11	1.53 × 10^16^	48	7	3.21 × 10^16^
4	86 330	4	5	1.24 × 10^15^	34	1	2.39 × 10^15^	66	1	3.63 × 10^15^
5	133 977	6	15	1.43 × 10^16^	44	9	1.86 × 10^16^	56	8	3.30 × 10^16^
6	114 532	5	6	1.11 × 10^16^	59	7	7.64 × 10^15^	41	3	1.87 × 10^16^
7^b^	16 399	1	0.8	4.42 × 10^14^	38	0	7.10 × 10^14^	62	0	1.15 × 10^15^
Total	2 235 673	100	188.8	1.53 × 10^17^	41	100	2.19 × 10^17^	59	100	3.72 × 10^17^
**After tourism adjustment**										
1	872 009	39	100	5.45 × 10^16^	50	38	5.45 × 10^16^	50	40	1.09 × 10^17^
2	757 418	34	42	4.55 × 10^16^	54	32	3.83 × 10^16^	46	28	8.39 × 10^16^
3	255 008	11	20	1.68 × 10^16^	52	12	1.53 × 10^16^	48	11	3.21 × 10^16^
4	86 330	4	5	1.24 × 10^15^	34	1	2.39 × 10^15^	66	2	3.63 × 10^15^
5	133 977	6	15	1.43 × 10^16^	44	10	1.86 × 10^16^	56	14	3.30 × 10^16^
6	114 532	5	6	1.11 × 10^16^	59	8	7.64 × 10^15^	41	6	1.87 × 10^16^
7	16 399	1	0.8	4.42 × 10^14^	38	0	7.10 × 10^14^	62	1	1.15 × 10^15^
Total	2 235 673	100	188.8	1.44 × 10^17^	51	100	1.37 × 10^17^	49	100	2.81 × 10^17^

Abbreviations: gc, gene copies; mg/d, million gallons per day.

^a^ Facility 1 receives flows from the Las Vegas Strip, and facilities 2 and 3 are the 2 most similar sewersheds in terms of population and infection burden. After the tourism adjustment, the relative site totals for facility 1 were more closely aligned with facilities 2 and 3 (ie, approximately 50% in each time period), and the relative viral loads by facility were more closely aligned with the population breakdown.

^b^ For period 1, facility 7 monitoring started on December 14, 2020 (ie, excludes first 3.5 months of time period).

**Figure 4.**
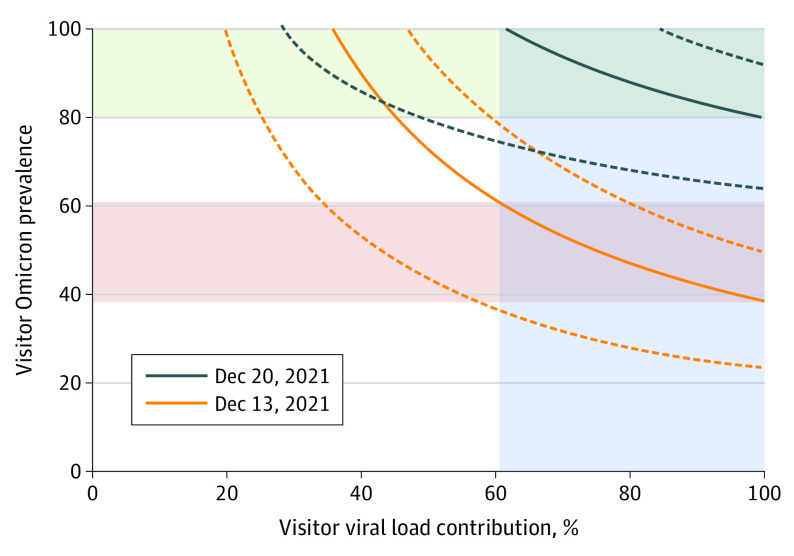
Estimated Omicron Prevalence Among Visitors vs Visitor Viral Load Contribution on December 13 and December 20, 2021 Solid lines represent potential solutions to the equation presented in the Statistical Analysis section based on mean Omicron prevalence from Figure 3, and dashed lines represent 90% CIs. Blue shading indicates the possible range for visitor viral load contribution (>60%), assuming the relative contribution was similar on both dates, and red and green shading indicate the corresponding range for Omicron prevalence among visitors on each date (40%-60% on December 13 and 80%-100% on December 20).

Leveraging paired sequencing data, we estimated that visitors contributed at least 60% of the viral load to facility 1 in December 2021, with Omicron prevalence among visitors ranging from approximately 40% to 60% on December 13 and from 80% to 100% on December 20 (Figure 4). These Omicron prevalence estimates are consistent with the Omicron single-nucleotide polymorphism frequencies observed in the Las Vegas Strip manhole on those same dates (48% and 82%, respectively) (Figure 3A). Interestingly, the US Centers for Disease Control and Prevention (CDC) initially reported that 73% of new COVID-19 cases in the United States were Omicron as of December 18 but subsequently revised this value downward to 23%.^13^ The WBE data from this study support the original CDC estimate, although visitors to Las Vegas are not necessarily representative of the US population as a whole, so the values may not be directly comparable.

After reducing facility 1’s viral load by 60% (ie, the estimate for visitor contributions), the relative viral loads for facilities 1 to 3 were more closely aligned with each other and with the sewershed population breakdown (Table). Moreover, the adjusted ascertainment ratio for facility 1 (mean [SD], 5.4 [1.9]) was also more consistent with that of facilities 2 and 3 (eTable 2 in Supplement 1). After adjustment, the mean (SD) ascertainment ratio for Facilities 1 to 3 was 4.6 (1.6), which is higher than the 3.4 (0.4) calculated earlier in the pandemic.^4^ This might be attributable to fewer individuals with infection seeking clinical testing, in part due to greater availability of at-home rapid antigen tests. Finally, using the adjusted viral load for facility 1 and the previously described framework for estimating COVID-19 incidence throughout southern Nevada,^4^ we estimated that approximately 476 000 local residents—approximately 20% of the population—contracted COVID-19 in January 2022 during the initial Omicron wave. In contrast, only 94 427 confirmed cases were reported for the 7 sewersheds during this same timeframe—an overall ascertainment ratio of 5.0.

## Discussion

Wastewater surveillance at municipal WWTPs can provide valuable information about local community transmission, but there are situations in which viral loads and VOC composition may be confounded by various factors. In this study, we found that visitors may have disproportionately contributed to wastewater surveillance data without being reflected in local clinical surveillance efforts. This can lead to overestimates when using wastewater SARS-CoV-2 concentrations to calculate infection burden. For example, the mean ascertainment ratio for facility 1 decreased from 14.0 to 5.4 after adjusting for contributions from visitors. Without this adjustment, the wastewater-derived infection estimate for this sewershed would be nearly 3-fold higher, leading to a highly inaccurate overall infection estimate for southern Nevada.

Detection of a visitor-contributed VOC through wastewater surveillance has the potential to erroneously suggest VOC circulation in the local community. On the other hand, it can also serve as an early warning signal for local transmission. In this study, Omicron was first detected in the Las Vegas Strip manhole 1 week prior to its detection at the WWTPs. Therefore, that initial detection may have been linked to a visitor rather than being indicative of community transmission, but it also foreshadowed the rapid onset of Omicron infections throughout southern Nevada.

### Limitations

This study has several limitations. The detection of SARS-CoV-2 RNA in wastewater does not indicate infectivity, so while a significant viral load may be contributed by visitors (>60% in this study), this does not necessarily prove that visitors are a significant contributor to local transmission. Studies show that individuals with infection can shed SARS-CoV-2 RNA in feces for several weeks after the onset of an infection,^14^ so visitors may contribute SARS-CoV-2 RNA to local sewersheds while in a recovering or recovered state. Therefore, analysis of wastewater surveillance data should be viewed only as a complement to clinical tools, but it can provide valuable, time-sensitive data for decision-makers and policy makers.

## Conclusions

The results of this cross-sectional study provide an estimate of the relative SARS-CoV-2 load from visitors to the Las Vegas Strip as a means of generating a more accurate estimate of COVID-19 incidence in the local population. Our wastewater results support the feasibility of understanding the potential confounding effects of mobile populations in WBE applications.

## Notes

Supplement 1. **eFigure.** New Daily Cases per 100 000 People and Corresponding Recovery-Adjusted Wastewater SARS-CoV-2 Concentrations for Facilities 1 to 7 From September 2021 through February 2022 **eTable 1.** Mutation Frequencies for the 19 Omicron-Specific Mutations Monitored in This Study, as a Function of Date and Sampling Location **eTable 2.** Comparison of Monthly Ascertainment Ratios for the Most Populous Sewersheds in Southern Nevada

Supplement 2.
Data Sharing Statement
