# Supplementary material for: Detection of the Omicron BA.1 Variant of SARS-CoV-2 in Wastewater From a Las Vegas Tourist Area
Source: JAMA Netw Open. 2023 Feb 23;6(2):e230550. doi: 10.1001/jamanetworkopen.2023.0550 (PMC9951036; doi:10.1001/jamanetworkopen.2023.0550)
Supplement: Supplement 1. — eFigure. New Daily Cases per 100 000 People and Corresponding Recovery-Adjusted Wastewater SARS-CoV-2 Concentrations for Facilities 1 to 7 From September 2021 through February 2022 eTable 1. Mutation Frequencies for the 19 Omicron-Specific Mutations Monitored in This Study, as a Function of Date and Sampling Location eTable 2. Comparison of Monthly Ascertainment Ratios for the Most Populous Sewersheds in Southern Nevada [file jamanetwopen-e230550-s001.pdf]

Supplemental Online Content

Vo V, Tillett RL, Papp K, et al. Detection of the Omicron BA.1 variant of SARS-CoV-2 in wastewater from a Las Vegas tourist area. *JAMA Netw Open.* 2023;6(2):e230550. doi:10.1001/jamanetworkopen.2023.0550

eFigure. New Daily Cases per 100 000 People and Corresponding Recovery-Adjusted Wastewater SARS-CoV-2 Concentrations for Facilities 1 to 7 From September 2021 through February 2022

eTable 1. Mutation Frequencies for the 19 Omicron-Specific Mutations Monitored in This Study, as a Function of Date and Sampling Location

eTable 2. Comparison of Monthly Ascertainment Ratios for the Most Populous Sewersheds in Southern Nevada

This supplemental material has been provided by the authors to give readers additional information about their work.

© 2023 Vo V et al. *JAMA Network Open.*

eFigure. New Daily Cases per 100 000 People and Corresponding Recovery-Adjusted Wastewater SARS-CoV-2 Concentrations for Facilities 1 to 7 From September 2021 through February 2022

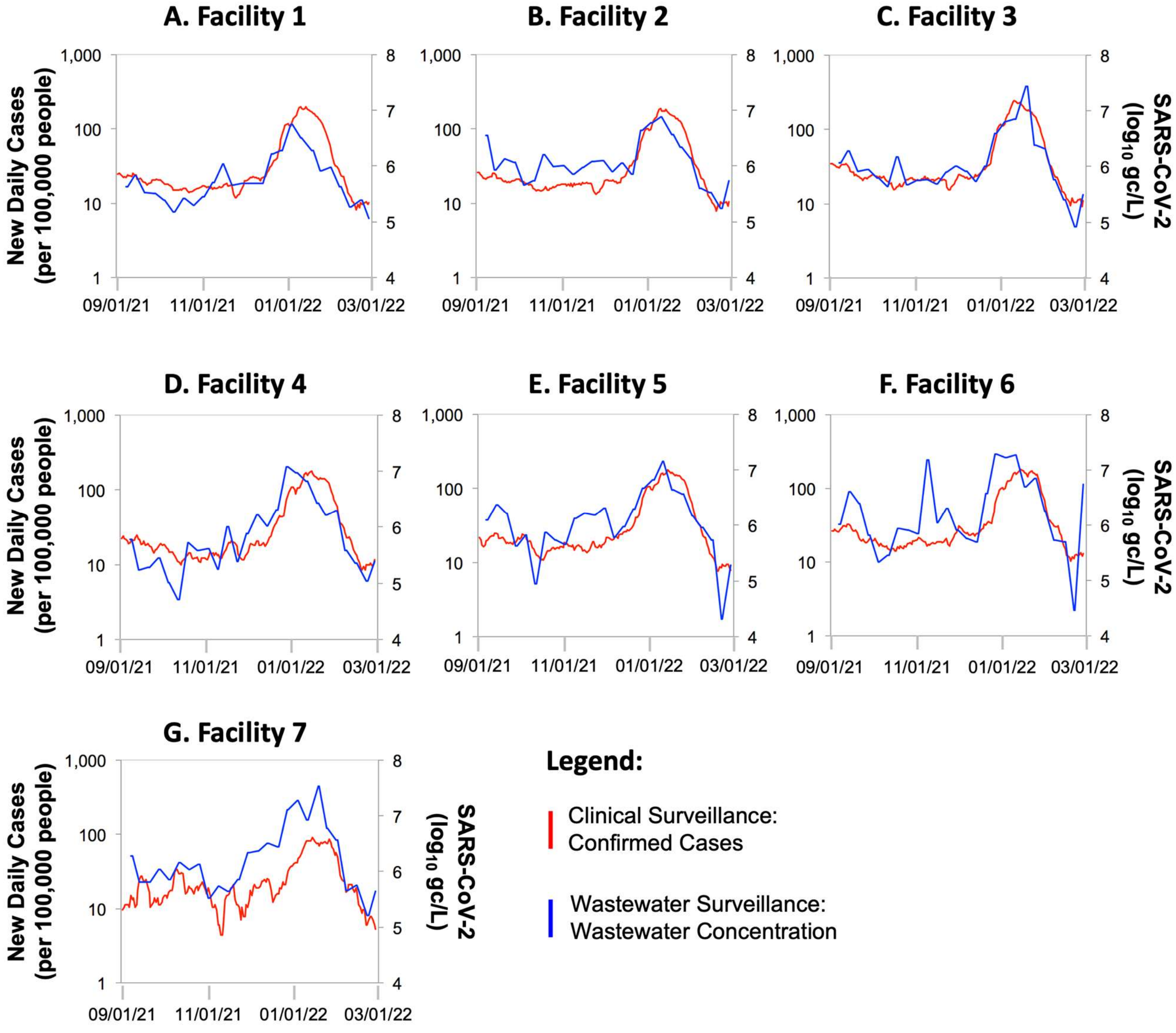

© 2023 Vo V et al. *JAMA Network Open.*

eTable 1. Mutation Frequencies for the 19 Omicron-Specific Mutations Monitored in This Study, as a Function of Date and Sampling Location

Because only a subset of the mutations was detected in the Dec 13th samples, and with highly variable frequencies, only the five mutations highlighted in yellow were used for calculating average frequencies. These were the only mutations consistently detected on that date across Facilities 1, 2, and 3, which were the facilities of interest for the viral load/Omicron prevalence analysis. For subsequent sampling dates (and for all sampling dates for the Las Vegas Strip manhole), all 19 mutations were detected in every sample but still at varying frequencies. For consistency, the average frequencies after Dec 13th still reflect only the five aforementioned mutations. Facility 7 is geographically isolated from Southern Nevada's major population center, which presumably explains its lower average frequencies on Dec 13th (non-detect) and Dec 20th.

12/13/21	Facility 1	Facility 2	Facility 3	Facility 2&3	Facility 4	Facility 5	Facility 6	Facility 7
M. A63T	22.91%	3.78%	--		5.05%	--	--	--
E. T9I	--	4.31%	--		--	--	--	--
ORF3a. Syn25584	--	--	--		6.25%	--	5.26%	--
S. syn25000	52.90%	4.94%	--		3.43%	--	--	--
S. L981F	58.33%	--	--		--	--	--	--
S. N969K	62.24%	--	--		--	--	--	--
S. N764K	--	--	--		4.10%	--	--	--
S. N679K	6.86%	--	--		3.68%	--	--	--
S. T547K	24.57%	4.65%	5.71%		6.74%	--	3.57%	--
S. S375F	41.44%	3.03%	3.57%		8.33%	--	--	--
S. S373P	41.67%	4.08%	3.57%		8.24%	--	--	--
S. S371L 2	43.40%	3.03%	3.57%		7.87%	--	--	--
S. S371L 1	41.12%	3.00%	7.14%		7.73%	--	--	--
S. G339D	23.26%	--	--		11.28%	--	--	--
S. ins214EPE	31.39%	--	--		3.60%	3.93%	--	--
S. del211/L212I	34.12%	--	--		3.98%	4.37%	--	--
ORF1b. I1566V	10.98%	--	--		3.80%	--	--	--
ORF1a. Syn13195	17.10%	--	7.14%		7.13%	--	--	--
ORF1a. P3395H	--	--	--		--	3.66%	--	--
**Average**	**38.44%**	**3.56%**	**4.71%**	**4.14%**	**7.78%**	**--**	**3.57%**	**--**
**Standard Deviation**	**7.80%**	**0.76%**	**1.64%**	**1.35%**	**0.64%**	**--**	**--**	**--**

12/20/21	Facility 1	Facility 2	Facility 3	Facility 2&3	Facility 4	Facility 5	Facility 6	Facility 7
M. A63T	44.31%	30.74%	26.94%		43.59%	24.85%	64.93%	19.89%
E. T9I	48.66%	34.66%	35.73%		57.81%	30.26%	70.91%	13.66%
ORF3a. Syn25584	56.67%	36.31%	30.76%		62.59%	30.17%	67.96%	20.75%
S. syn25000	74.57%	31.01%	24.75%		51.91%	27.15%	59.86%	13.62%
S. L981F	50.81%	29.53%	27.50%		65.06%	36.87%	57.96%	14.18%
S. N969K	50.93%	30.68%	32.50%		65.20%	36.81%	57.69%	14.23%
S. N764K	60.08%	32.08%	25.93%		57.81%	31.75%	66.17%	20.59%
S. N679K	58.13%	41.79%	37.71%		56.95%	38.01%	69.59%	18.36%
S. T547K	62.98%	53.12%	44.42%		60.38%	42.40%	77.42%	12.64%
S. S375F	84.00%	49.26%	45.28%		61.45%	40.24%	71.47%	28.40%
S. S373P	84.16%	49.33%	45.40%		61.65%	40.49%	71.99%	28.45%
S. S371L 2	84.31%	49.19%	45.81%		61.73%	40.14%	72.23%	28.51%
S. S371L 1	84.24%	49.48%	45.56%		61.66%	39.97%	71.96%	28.42%
S. G339D	86.70%	50.79%	42.06%		63.87%	42.47%	71.23%	18.78%
S. ins214EPE	69.19%	49.16%	49.43%		51.11%	44.44%	77.77%	23.30%
S. del211/L212I	70.76%	50.50%	51.12%		53.39%	45.40%	79.56%	23.69%
ORF1b. I1566V	73.69%	32.56%	34.84%		53.80%	19.97%	66.99%	13.62%
ORF1a. Syn13195	68.31%	40.14%	39.81%		63.56%	32.09%	71.71%	24.13%
ORF1a. P3395H	53.64%	37.01%	36.33%		48.75%	37.80%	68.50%	14.22%
**Average**	**79.94%**	**50.08%**	**45.29%**	**47.68%**	**61.37%**	**40.65%**	**73.02%**	**25.28%**
**Standard Deviation**	**9.48%**	**1.71%**	**0.53%**	**2.79%**	**0.57%**	**1.00%**	**2.48%**	**7.07%**

eTable 1 (continued). Mutation Frequencies for the 19 Omicron-Specific Mutations Monitored in This Study, as a Function of Date and Sampling Location

12/27/21	Facility 1	Facility 2	Facility 3	Facility 2&3	Facility 4	Facility 5	Facility 6	Facility 7
M. A63T	86.29%	79.46%	78.88%		91.73%	84.36%	94.70%	90.94%
E. T9I	92.76%	84.32%	84.23%		94.90%	85.72%	96.07%	93.05%
ORF3a. Syn25584	91.17%	82.69%	88.11%		95.80%	86.64%	95.37%	92.74%
S. syn25000	86.56%	78.46%	75.82%		94.19%	80.85%	92.47%	91.34%
S. L981F	57.78%	76.09%	79.29%		95.27%	81.77%	91.75%	90.01%
S. N969K	65.82%	76.17%	79.40%		95.69%	82.19%	91.99%	90.38%
S. N764K	93.59%	81.70%	81.82%		92.43%	83.37%	93.07%	92.25%
S. N679K	91.73%	80.36%	85.32%		92.13%	86.84%	94.52%	93.29%
S. T547K	94.68%	85.68%	87.12%		95.27%	90.85%	96.39%	95.09%
S. S375F	94.27%	78.49%	88.55%		93.04%	87.39%	94.78%	93.69%
S. S373P	94.44%	78.92%	88.91%		93.10%	87.66%	95.12%	94.17%
S. S371L 2	94.11%	78.93%	88.82%		93.03%	87.53%	95.01%	93.86%
S. S371L 1	94.27%	78.97%	88.92%		93.20%	87.48%	95.12%	94.19%
S. G339D	93.40%	84.29%	90.80%		94.88%	87.60%	94.95%	94.23%
S. ins214EPE	92.31%	83.52%	88.50%		92.59%	83.31%	92.73%	92.16%
S. del211/L212I	94.46%	85.97%	91.37%		94.50%	87.14%	95.34%	95.04%
ORF1b. I1566V	85.43%	75.43%	79.60%		90.40%	84.22%	94.36%	92.84%
ORF1a. Syn13195	89.13%	81.97%	85.96%		94.97%	84.41%	95.01%	94.02%
ORF1a. P3395H	90.25%	76.37%	81.44%		92.80%	80.28%	93.47%	91.39%
**Average**	**94.35%**	**80.20%**	**88.46%**	**84.33%**	**93.53%**	**88.18%**	**95.28%**	**94.20%**
**Standard Deviation**	**0.22%**	**3.07%**	**0.76%**	**4.84%**	**0.98%**	**1.49%**	**0.63%**	**0.54%**

Las Vegas Strip	12/7/21	12/13/21	12/20/21	12/26/21
M. A63T	12.90%	37.68%	73.42%	84.62%
E. T9I	8.53%	56.63%	76.16%	86.90%
ORF3a. Syn25584	12.99%	43.01%	76.77%	88.41%
S. syn25000	10.64%	47.89%	71.13%	81.33%
S. L981F	8.01%	48.00%	74.97%	77.94%
S. N969K	9.03%	46.56%	74.98%	82.47%
S. N764K	13.11%	45.23%	72.13%	80.92%
S. N679K	6.22%	48.24%	80.51%	85.29%
S. T547K	15.27%	55.32%	87.59%	90.29%
S. S375F	10.53%	46.45%	80.72%	91.63%
S. S373P	9.88%	45.81%	80.72%	92.02%
S. S371L 2	10.81%	46.05%	81.12%	92.02%
S. S371L 1	10.40%	45.91%	80.78%	92.02%
S. G339D	20.16%	58.73%	86.80%	87.33%
S. ins214EPE	26.62%	53.59%	83.88%	86.37%
S. del211/L212I	29.10%	58.28%	85.41%	88.62%
ORF1b. I1566V	6.64%	52.29%	69.53%	82.82%
ORF1a. Syn13195	12.36%	53.98%	79.78%	85.90%
ORF1a. P3395H	8.87%	43.29%	77.19%	82.94%
**Average**	**11.38%**	**47.91%**	**82.19%**	**91.59%**
**Standard Deviation**	**2.20%**	**4.15%**	**3.02%**	**0.75%**

© 2023 Vo V et al. *JAMA Network Open.*

eTable 2. Comparison of Monthly Ascertainment Ratios for the Most Populous Sewersheds in Southern Nevada

Ascertainment ratios for Facility 1 are shown without and with the adjustment for visitor contributions.

Month	Facility 1 (No Adjustment)	Facility 1 (With Adjustment)	Facility 2	Facility 3
September 2021	13	5	6	5
October 2021	8	3	4	4
November 2021	23	9	6	4
December 2021	12	5	2	3
January 2022	13	5	3	6
February 2022	13	5	3	4
**Average**	**14 ± 4.8**	**5.4 ± 1.9**	**4.2 ± 1.4**	**4.5 ± 1.2**

© 2023 Vo V et al. *JAMA Network Open.*
