# Supplementary material for: Detection of the Omicron BA.1 Variant of SARS-CoV-2 in Wastewater From a Las Vegas Tourist Area
Source: JAMA Netw Open. 2023 Feb 23;6(2):e230550. doi: 10.1001/jamanetworkopen.2023.0550 (PMC9951036; doi:10.1001/jamanetworkopen.2023.0550)
Supplement: Supplement 2. — Data Sharing Statement [file jamanetwopen-e230550-s002.pdf]

# Data Sharing Statement

Vo. Detection of the Omicron BA.1 Variant of SARS-CoV-2 in Wastewater From a Las Vegas Tourist Area. *JAMA Netw Open*. Published February 23, 2023.
doi:10.1001/jamanetworkopen.2023.0550

**Data**
**Data available:** Yes
**Data types:** Data (not involving human participants)
**How to access data:** Edwin.oh@unlv.edu
**When available:** With publication

**Supporting Documents**
**Document types:** None

**Additional Information**
**Who can access the data:** anyone requesting the data
**Types of analyses:** sequencing data
**Mechanisms of data availability:** with investigator support
